# The Potential of Hyperspectral Patterns of Winter Wheat to Detect Changes in Soil Microbial Community Composition

**DOI:** 10.3389/fpls.2016.00759

**Published:** 2016-06-09

**Authors:** Sabrina Carvalho, Wim H. van der Putten, W. H. G. Hol

**Affiliations:** ^1^Department of Terrestrial Ecology, NIOO–KNAW, Netherlands Institute of EcologyWageningen, Netherlands; ^2^Laboratory of Nematology, Wageningen UniversityWageningen, Netherlands

**Keywords:** *Triticum aestivum* L., serial dilution, land use, biodiversity, species loss, monitoring

## Abstract

Reliable information on soil status and crop health is crucial for detecting and mitigating disasters like pollution or minimizing impact from soil-borne diseases. While infestation with an aggressive soil pathogen can be detected via reflected light spectra, it is unknown to what extent hyperspectral reflectance could be used to detect overall changes in soil biodiversity. We tested the hypotheses that spectra can be used to (1) separate plants growing with microbial communities from different farms; (2) to separate plants growing in different microbial communities due to different land use; and (3) separate plants according to microbial species loss. We measured hyperspectral reflectance patterns of winter wheat plants growing in sterilized soils inoculated with microbial suspensions under controlled conditions. Microbial communities varied due to geographical distance, land use and microbial species loss caused by serial dilution. After 3 months of growth in the presence of microbes from the two different farms plant hyperspectral reflectance patterns differed significantly from each other, while within farms the effects of land use via microbes on plant reflectance spectra were weak. Species loss via dilution on the other hand affected a number of spectral indices for some of the soils. Spectral reflectance can be indicative of differences in microbial communities, with the Renormalized Difference Vegetation Index the most common responding index. Also, a positive correlation was found between the Normalized Difference Vegetation Index and the bacterial species richness, which suggests that plants perform better with higher microbial diversity. There is considerable variation between the soil origins and currently it is not possible yet to make sufficient reliable predictions about the soil microbial community based on the spectral reflectance. We conclude that measuring plant hyperspectral reflectance has potential for detecting changes in microbial communities yet due to its sensitivity high replication is necessary and a strict sampling design to exclude other ‘noise’ factors.

## Introduction

World food production relies heavily on soils, which are suffering from (human-induced) threats like loss of biodiversity due to intensified agriculture (Tsiafouli et al., 2015). Reliable information on soil status can be crucial for detecting and minimizing impact on crops from soil diseases. Remote sensing is a potentially powerful non-destructive method for obtaining information on crop health; reflected light spectra can be used as early warning for fungal diseases in olives (Mahlein et al., 2012; Calderon et al., 2013) and infestations of bark beetles in forest (Lausch et al., 2013). Therefore the technique has proven useful for detecting certain pests and diseases attacking aboveground plant parts, and arguably this can be adopted for a wider variety of targets including aﬄictions of the belowground part (Carvalho et al., 2012). While it might be relatively easy to detect infestation with an aggressive pathogen, it is unknown to what extent hyperspectral reflectance could be used to detect more subtle changes in a complex soil community. The belowground microbial community associated with plant roots is highly diverse and hyperspectral reflectance could be one of the new tools to identify how plants respond to shifts in soil community composition (Powell et al., 2013). It has also been suggested that remote sensing of vegetation could be used to study the spatial distribution and dynamics of soil microbial communities (Hamada et al., 2014).

Intensive agriculture is one important factor responsible for reduced biodiversity in soils (Tsiafouli et al., 2015). Changes in soil biota are important to monitor and detect early, since loss of soil biodiversity translates into loss of ecosystem services (de Vries et al., 2013). Early detection of belowground species loss could be used to adapt current soil management in order to prevent further loss of soil biodiversity and ecosystem functioning. While loss of larger organisms like earthworms might be relatively easy to observe, loss of microbes, such as bacteria will require specialized methods, which can be cultivation-based or molecular approaches. Here we want to explore the potential of hyperspectral reflectance of plants as an alternative to detect bacterial species loss from soil.

Bacterial communities show high spatial variation at continental scale, and this variation is partly related to ecosystem type and pH (Fierer and Jackson, 2006). Regional variation in soil type, pH and climate is expected to result in different microbial communities, while within a region management like plowing and fertilization may further modify microbial communities (Zhalnina et al., 2013). At small scales variation in microbial communities is still expected due to soil heterogeneity and dispersal limitations of microorganisms (Yergeau et al., 2010; Martiny et al., 2011). It remains an open question whether microbial species loss can be detected against this background of variation in microbial soil communities across spatial scales.

Hyperspectral reflectance has been used to assess the extremes of the soil biota spectrum represented by presence or absence of soil biota (Carvalho et al., 2012). On basis of their hyperspectral reflectance patterns plants that were growing in sterilized soils could be discriminated from plants growing in sterilized soils inoculated with live soil with 50–60% correctness (Carvalho et al., 2012). Live soil contains a variety of organisms, large ones like earthworms, ants, and larvae and microbial ones like protozoa, bacteria, and fungi. Some of those organisms will affect plant chemical composition, either by mutualistic effects such as providing nutrients, producing growth hormones, suppressing diseases (Hol et al., 2014) or by pathogenic interactions, which damage plant tissue and could trigger the plants’ defense system (Termorshuizen, 2014). Variation in the plant’s chemical composition can be detected by high-resolution spectroscopy (Asner and Martin, 2008; Ramoelo et al., 2012; Carvalho et al., 2013a,b; Kokaly and Skidmore, 2015). At the field scale it has been found that the addition of nitrogen significantly affected spectral parameters, while addition of endophytic bacteria did not affect the spectra of winter wheat plants (Adami et al., 2010). In the present study we focus on the microbial organisms (<45 μm) in soil, and test to what extent hyperspectral reflectance of winter wheat plants will be influenced by differences in soil microbial community composition.

We examined how changes in microbial community composition will alter hyperspectral reflectance of plants. We used winter wheat grown in a greenhouse experiment established to assess effects of microbial species loss on plant productivity (Hol et al., 2015a). Winter wheat (*Triticum aestivum* L.) was selected as it is a common crop of global importance, and because it has been used in remote sensing studies on effects of addition of nitrogen and endophytic bacteria (Adami et al., 2010), or pests and diseases (Yuan et al., 2014). We wanted to test if the method of hyperspectral reflectance from a single leaf is sensitive enough to detect changes in microbial community composition, provided everything else is standardized.

Growing plants in sterilized soil with microbial inocula allowed us to focus on the effect of variations in microbial communities. Exclusion of larger soil organisms, a uniform sterilized background soil for all treatments, and environmental controlled conditions created suitable test scenarios. Additional to the samples’ variation caused by geographical distance and land use, we super-imposed species loss by using dilution-to-extinction in the microbial inocula. Serial dilution of inoculum will reduce less abundant species while dominant species remain present in all treatments and thus could be considered as a relatively subtle change in species composition while species richness declines. This method has been used before to successfully manipulate microbial community composition and determine effects on soil processes such as decomposition and plant growth promotion (Griffiths et al., 2001; Wertz et al., 2006, 2007; Hol et al., 2010, 2015a,b; Baumann et al., 2013; Tardy et al., 2014).

Our main question was: can hyperspectral reflectance of plants be used to detect differences in microbial community composition? We tested the hypotheses that spectra can be used to (1) separate plants growing with microbial communities from different farms; (2) to separate plants growing in different microbial communities due to different land use; and (3) separate plants according to microbial species loss.

## Materials and Methods

### Greenhouse Experiment

The experiment was established in 2009 and the setup is described in detail in Hol et al. (2015a). Briefly, soil was collected from two Swedish farms, sampling 3 fields per farm and collecting two samples per field, separated by at least 30 m. This resulted in 12 soil origins (2 farms × 3 land use types × 2 replicates per field). The farms were separated by 25 km distance. Within a farm the maximum distance between fields with different land use was 3 km. The three land use types per farm were grassland (G), and intensive monoculture (I) and an extensive crop rotation (R). Field soil properties have been reported before (de Vries et al., 2013, Supplementary Material). Soil from those fields was sterilized (25 kGy) and inoculated with diluted microbial suspensions according to Hol et al. (2010, 2015a). Briefly, the suspension was obtained by mixing soil with water in a blender, followed by centrifugation to remove soil particles. The suspension was filtered over 45 μm to exclude meso and macrofauna. For each of the 12 soil origins three dilution treatments were created (10^-2^, 10^-4^, and 10^-6^). Essentially, the highest inoculum level was 5 kg sterile soil inoculated with a microbial suspension obtained from 25 g of field soil. After addition of the suspension to the sterilized soils, the bags were shaken to mix the suspension; this mixing was repeated biweekly. The inoculated soils were incubated in the dark at room temperature for 8 months in order to allow the microorganisms to fully colonize the soil. After the incubation period 24 pots per treatment were filled with soil and those soils were sown with *T. aestivum* L. cv. Carenius seeds. The pots were arranged in 24 blocks (fully randomized block design) in order to account for spatial effects in the greenhouse.

The plants were grown for 8 weeks, allowing plant-dependent microbes to also fully colonize the soil. At the end of this first growing period 50 g fresh weight soil from each pot was stored at 4°C to be used in the second growing phase. All remaining soil was pooled, homogenized and sterilized by gamma radiation (25 kGy) to establish a uniform background soil. Sterilization by gamma radiation at the applied dose should be effective in killing most microorganisms (McNamara et al., 2003) and may enhance availability of nitrogen and phosphate (Troelstra et al., 2001). Nevertheless, all our treatments were started from sterilized soil, so that nutrient release and potential radio-resistant organisms should be similar among dilution treatments and thus not can explain differences between treatments. Note that the experimental soils are not comparable to the field situation; our aim was to test whether differences in microbial communities can be detected by means of hyperspectral reflectance from plants. The stored soil from the first growing phase was used as 20% inoculum: all pots contained 200 g sterilized soil fully mixed with 50 g living soil as inoculum. Pots were sown with seeds from *T. aestivum* L. cv. Carenius. Growing conditions were 60% relative humidity; 16 h L, 8 h D, 21°C/16°C, and additional illumination by 400 W growing bulbs (Philips SONT-T Agro, Philips, Eindhoven, Netherlands). Light intensity at plant level was 225 μmol PAR. In September a spontaneous aphid infestation of *Rhopalosiphum padi* L. occurred and all plants became infested. Six weeks after germination the leaf spectra were measured, as described in the next paragraph. After a growing period of 9 weeks in total, shoot biomass of plants was harvested by clipping at the soil surface and drying at 70°C until constant weight. For a subset of samples (*n* = 112) the percentage of nitrogen in the leaves was measured to test its correlation with the spectral indices. Nitrogen was measured in two milligrams of the dried, ground leaf material, by combustion with an elemental autoanalyzer Flash EA 1112 NC analyzer (Interscience, Breda, Netherlands). The bacterial community in a subset of the pots (*n* = 36; 2 origins × 3 dilutions × 6 blocks) was assessed by multiplex pyrosequencing in order to verify the effectiveness of the dilution treatment in reducing bacterial diversity. Dilution will most likely also have affected other microbes, such as archaea, fungi and protozoa. We therefore refer to effects of dilution as effects of microbial species loss and use the bacterial analysis to validate that dilution causes species loss. We selected pots from two soil origins which showed contrasting reactions in shoot biomass to dilution in the first phase of the experiment (Hol et al., 2015a). Directly after harvesting the shoot biomass, 2 g soil was collected from each pot at ∼5 cm depth and stored at -20°C until extraction. DNA was extracted from 0.25 g of with the Mobio Powersoil DNA extraction kit and amplified with the primer set 515F-806R (Lauber et al., 2009). Samples were sequenced on a Roche 454 automated sequencer and GS FLX system using titanium chemistry (454 Life Sciences, Branford, CT, USA) (Macrogen Inc. Company, South Korea). Dataprocessing was done as described in Hol et al. (2015a). As estimate for bacterial diversity the expected species richness was calculated for a sample size of 1440 reads with the vegan package in R (R Core Team, 2015).

### Leaf Spectral Measurements and Processing

To collect the spectral reflectance data in the visible and infrared region of the electromagnetic radiation (350–500) a plant probe with leaf-clip attached to an ASD Fieldspec three fieldspectrometer (ASD inc., Boulder CO, USA, henceforth ASD fieldspec) was used. The resolution of the ASD fieldspec 3 is of 3 nm in the 350 – 1000 nm, and 10 nm between 1000 and 2500 nm wavelengths. The light bulb used in the probe was heat sensitive halogen of color and temperature 2901 ± 10% K. The radius of the spectral measurement was of 10 mm. We measured the spectral reflectance patterns of the first fully developed leaf of 6-week-old winter wheat plants with the black panel face of the probe as background. The calibration was done with the white reference face of the leaf-clip. As the winter wheat leaves were narrower than the plant probe standard radius the measured leaf was always fixed in the central area in the vertical position. All measurements were offset corrected and the noisy region between 350 and 400 nm eliminated using the software ViewSpec Pro 5.6.10 (ASD inc. Boulder, USA).

Hyperspectral indices (referred to as spectral indices throughout the paper) were calculated in order to evaluate the differences in plants due to chlorophyll content (NDVI), nitrogen (NRI), plant physiognomy (EVI), water limitation or water-stress (WI, DSWI), nutrient and photosynthesis stress (PS, REP), photosynthetic shift (ARI), and plant senescing (PSRI) among others (**Table 1**).

**Table 1:** Overview of the indices used in the manuscript to assess the effect of microbial communities on reflectance.

Code	Definition	Formula	Description	References
NRI	Nitrogen reflection index	(R_570_–R_670_)/ (R_570_+R_670_)	Relates to nitrogen content	Filella et al., 1995
NDVI	Normalized difference vegetation index (NDVI)	(R_800_–R_670_)/ (R_800_+R_670_)	Chlorophyll content	Tucker, 1979
RDVI	Re-normalized difference vegetation index	(R_805_–R_710_)/ √(R_805_+R_657_)	Sensitive to chlorophyll and nitrogen	Ramoelo et al., 2012
EVI	Enhanced vegetation index (EVI)	2.5^∗^(R_800_–R_670_)/ (R_800_+(6^∗^R_670_) – (7.5^∗^R_475_)+1)	Plant physiognomy	Huete et al., 1997
REP	Red-edge position	R_700_+40^∗^(((R_670_+ R_780_)/2–R_700_)/(R_740_–R_700_))	Nutrient or general plant stress	Clevers et al., 2002
mREP	Modified red-edge position	(R_750_–R_705_)/ (R_750_+R_705_ – 2^∗^R_445_)	Nutrient or general plant stress	Sims and Gamon, 2002
PRIa	Photosynthetic radiation index	(R_531_–R_570_)/ (R_531_+R_570_)	Pigment stress especially Xantophylls due to band 531nm	Gamon et al., 1992
WI	Water index	(R_900_/R_970_)	Water content	Apan et al., 2004
DSWI	Disease-water stress index	(R_800_/R_1660_)	Related water content changes due to plant diseases	Hamzeh et al., 2013
PSa	Plant stress index	(R_695_/R_420_)	Plant stress	Carter, 1994
ARI	Anthocyanin reflectance index	(1/R_550_) – (1/R_700_)	Pigment stress especially Anthocyanin	Gitelson et al., 2001
PSRI	Plant senescence reflectance index	(R_680_–R_500_)/R_750_	Plant senescence indicator due to photosynthesis shift	Merzlyak et al., 1999

*Shown are the abbreviated codes, their names, formulas, descriptions and references.*

### Data-analysis

Linear discriminant analysis (LDA) was applied into the full spectral range to find discriminant functions that best explain the relationship between the variables we have and the groups that we are interested in (i.e., farm, intensity, or dilution). The discrimination of intensity and dilution treatments was performed within each farm to disentangle it from the farm effect. The LDA functions were built using 70% of the samples as a training-cross-validation dataset, while 30% was reserved as testing/predicting set. To avoid spectral collinearity LDA was performed using Principal Component Analyses (PCA) scores. We selected the first 20 PCs and Mahalanobis distance as the distance measure for group discrimination. To reduce the impact of different group sizes the prior probabilities were considered (Naes et al., 2002; Quinn and Keough, 2002). The testing set allows us to evaluate the LDA success to correctly classify unknown samples. The better the functions are the better the classification of unknown samples into the groups defined. This procedure was done in Unscrambler X 10.1 and SPSS 20.0 for Windows.

We tested the effect of microbial origin (farm and land use) on spectral indices by doing ANOVAs on the averaged values per field. For each field the measurements were averaged across blocks and dilution treatments to remove pseudoreplication, resulting in six datapoints per farm. Since testing multiple indices would enhance the chance of Type I errors, we used false discovery rate control via the sharpened Benjamini Hochberg procedure (Verhoeven et al., 2005). Additionally, we tested whether there was a significant effect of dilution on a selection of indices for each of the 12 soil origins separately (**Table 1**). Linear mixed effect models (LMEs) were used with block as random factor: model1 ← lme(index∼dilution, random = ∼1| block, method = “ML”). Residuals did not always meet assumptions of normality. In order to avoid false positives due to this lack of normality, all tests resulting in *P* < 0.05 were repeated with non-parametric tests. In those tests blocks were always included to account for spatial variation in the greenhouse. The Prentice test was used, which is a generalized Friedman rank sum test. For paired comparisons between two treatments Wilcoxon’s signed rank test was used. Correlations between shoot biomass and spectral indices, or between bacterial species richness and spectral indices were statistically tested with Spearman rank correlations. The analyses were done in R 3.0.3 (R Core Team, 2015) using the ‘nlme’ package (Pinheiro et al., 2015) and Prentice tests with the ‘muStat’ package (Wittkowski and Song, 2012). All data presented here are available via Dryad (Hol et al., 2016).

## Results

### Spectra Related to Farms

**FIGURE 1:**
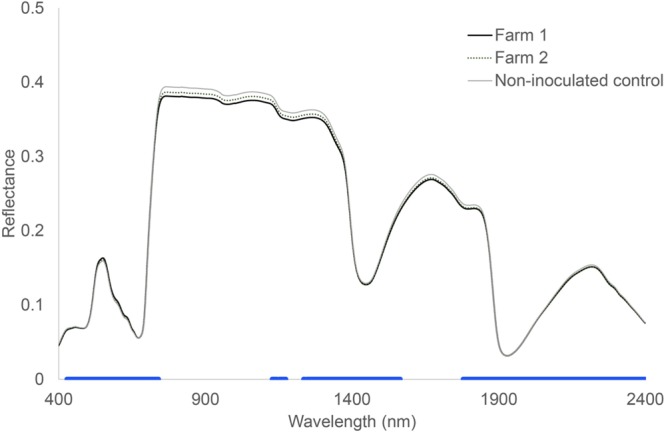
**Hyperspectral reflectance of *Triticum aestivum* L. plants growing in soils inoculated with microorganisms from two different farms.** The blue bar at the bottom indicates where there were no significant differences between control soil and both the inoculated soils. Wilcoxon test, *P* < 0.05, *n* = 24.

**FIGURE 2:**
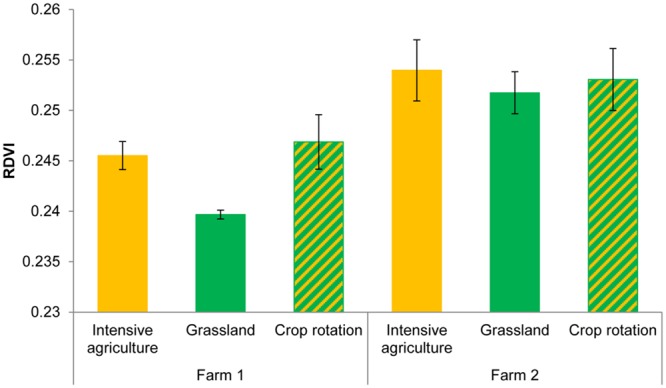
**RDVI of *Triticum aestivum* L. plants growing in the presence of the microorganisms from two farms with three land use types.** ANOVA Farm *F* = 23.08, df_1,8_ *P* = 0.001; Field *F* = 2.23, df_2,8_ *P* = 0.17.

Hyperspectral reflectance clearly differed between plants growing in sterilized soils and plants growing in sterilized soil inoculated with microorganisms from two farms (**Figure 1**). Most spectral indices (NDVIc, RDVI, REP, mREP, PRIa, WI, DSWI, PSa) were significantly different between microbial inocula from the two farms. The lowest values were found in Farm 1, independent of land use (**Figure 2**). The farm LDA using the training set showed that the two discriminant functions explained 70.6 and 19.9% of the variance, respectively. The combination of the two LDA functions could significantly discriminate between the farms (**Table 2A**, *P* < 0.001). Despite the significant difference in spectral indices between plants growing with microorganisms from the two farms, no significant overall differences in shoot biomass could be detected (data not shown). The spectra appear to reflect qualitative rather than quantitative differences. Our indicator of plant quality, percentage of nitrogen in the leaf, correlated weakly with NRI (ρ -0.227, *P* < 0.05, Spearman rank correlation test).

**Table 2:** Linear discriminant analyses confusion matrices for training/cross-validation and test of unknown samples.

(A)	Farms	1	2	C						
Training	1	**174**	70	4						
	2	97	**222**	13						
	Control	0	0	**14**						
Test	1	**39**	35	3						
	2	52	**62**	8						
	C	0	0	**0**						

**(B)**	**Farm 1**									

	**Intensity**	Int	Grass	Rot	C	**Dilution**	3	2	1	C
Training	Int	**53**	7	3	1	3	**52**	5	13	4
	Grass	16	**73**	19	8	2	12	**72**	14	2
	Rot	17	17	**66**	4	1	22	18	**63**	7
	Control	0	0	0	**19**	Control	0	0	0	**19**
Test	Int	**5**	7	7	3	3	**4**	5	9	0
	Grass	16	**13**	13	2	2	15	**11**	10	6
	Rot	7	12	**9**	5	1	10	14	**11**	3
	Control	1	1	0	**0**	Control	0	0	1	**1**

**(C)**	**Farm 2**									

	**Intensity**	Int	Grass	Rot	C	**Dilution**	3	2	1	C
Training	Int	**81**	32	25	12	3	**53**	18	9	7
	Grass	8	**57**	13	3	2	8	**50**	5	2
	Rot	7	10	**58**	4	1	33	32	**83**	8
	Control	0	0	0	**13**	Control	0	0	0	**15**
Test	Int	**23**	14	24	4	3	**14**	8	12	1
	Grass	7	**11**	8	4	2	3	**4**	6	1
	Rot	2	7	**1**	1	1	1	20	**15**	7
	Control	1	0	0	**1**	Control	0	1	0	**0**

***(A)** LDA between samples grown in inoculated soil of the 2 farms and control (sterilized soil); **(B)** LDA for intensities or dilution treatments within farm 1 and **(C)** LDA for intensities or dilution treatments within farm 2. With exception of farm2 dilution treatments all LDA were significant considering the maximum number of discriminant functions (*P* < 0.02). Rows represent the predicted group and columns the actual group. The bold numbers along the diagonal represent the number of correctly classified samples.*

### Spectra Related to Different Land Use

Within each farm soil samples were collected from 3 different types of land use to serve as inocula in the greenhouse experiment. Analyzed for both farms together this did not yield any significant difference in spectral reflectance, and therefore there is no evidence of a general effect of land use on spectra. The LDA for intensity of land use within farm showed that all three functions together were significantly explaining the variation in the underlying dimensions [**Tables 2B,C**; *P*(Farm 1 land use) = 0.003, *P*(Farm 2 land use) = 0.016)]. The first LDA function explained approximately 60% of the variance while the second 20% of the variance and the third >12% of the variance.

### Detecting Microbial Species Loss

For about half the 12 soil origins a significant difference in spectral reflectance between dilution treatments was found, with spectral indices being more responding than individual wavelengths (**Table 3**). The attempted classification of the dilution groups revealed that only the LDA within farm 1 was statistically significant (*P* = 0.001) with 65% of the variance explained by the first function while the second explained 19.6% and the third 15% of the variance. Independent of the training LDA analyzed, the function plot (data not shown) showed the first discriminant function always separated control (not inoculated) samples from the other groups. The second function alone failed to differentiate groups (*P* > 0.05), revealing that disentangling the other treatment groups is difficult due to dimension overlap. This is supported by the outcome of the confusion matrix where the training set showed high classification of true positives but the test confusion matrices showed that correct classification of unknown samples was low (**Tables 2B,C**). The loadings inspection showed that the visible spectral region and red-edge slope were the regions that most contributed to the groups separation.

**Table 3:** Wavelengths and indices which showed significant differences according to dilution treatment.

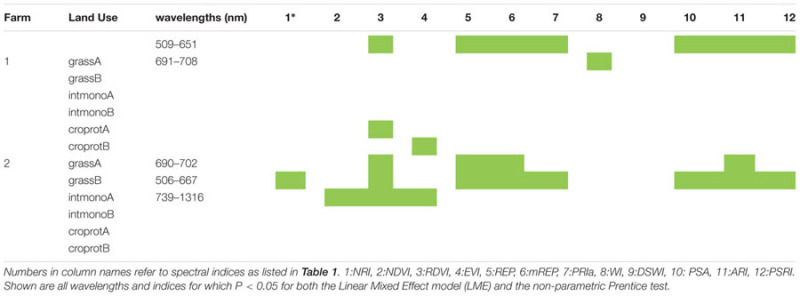

**FIGURE 3:**
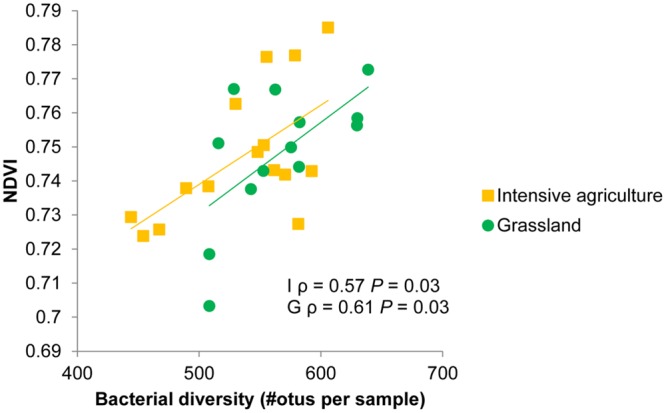
**Normalized difference vegetation index (NDVI) values of winter wheat plants growing in soils with different bacterial composition as a consequence of soil dilution treatments.** Bacterial diversity is measured as the number of different operational taxonomic units (otus) per 1440 reads. Spearman rank correlation coefficients (ρ) and *p*-values (*P*) are shown for an intensive (I) and grassland (G) soil from farm 2. See **Table 4** for correlations between bacterial species richness and other spectral indices.

The subset of data for which information was available on bacterial community composition showed a positive relation between spectral reflectance and bacterial species richness. A range of wavelengths (725–1382 nm and 1518–1853 nm) correlated with species richness of bacteria in soil, resulting in significant positive correlations between bacterial species richness and the normalized difference vegetation index (NDVI) and the enhanced vegetation index (EVI) (**Table 4**). The effect of bacterial species richness on spectral indices was still significant in a model where first soil origin and dilution were fitted as factors. The samples in the sequencing dataset originated from two different fields from the same farm, and also when the correlations were calculated separately per field they showed the same positive correlation between species richness of bacteria in soil and indices as the combined datasets (**Table 4**; **Figure 3**). For the intensive field NDVI and EVI had comparable significant correlations with bacterial species richness, while for the grassland soil NDVI showed higher dependence (ρ) than EVI on bacterial species richness.

**Table 4:** Bacterial species richness correlates positively with certain hyperspectral indices.

	Both fields		Intensive		Grassland	
	ρ	*P*	ρ	*P*	ρ	*P*
NRI	0.243	0.212	0.454	0.092	0.143	0.643
NDVI	0.588	**0.001**	0.571	**0.029**	0.615	**0.029**
RDVI	0.354	0.066	0.364	0.182	0.225	0.459
EVI	0.507	**0.006**	0.536	**0.042**	0.385	0.196
REP	0.043	0.829	0.004	0.995	-0.038	0.906
mREP	-0.041	0.838	-0.068	0.812	-0.038	0.906
PRIa	0.248	0.203	-0.146	0.602	0.505	0.081
WI	-0.079	0.687	-0.054	0.853	-0.016	0.964
DSWI	0.042	0.833	-0.186	0.507	0.071	0.821
PSa	0.131	0.505	0.339	0.216	0.104	0.737
ARI	0.195	0.318	0.457	0.089	0.011	0.978
PSRI	-0.055	0.780	0.004	0.995	-0.022	0.949

*Spearman rank correlations n = 15 for intensive monoculture, *n* = 13 for grassland, both from farm 2. Calculation of indices is described in **Table 1**. Bold numbers indicate significant *p*-values after false discovery rate control via the sharpened Benjamini Hochberg procedure.*

**FIGURE 4:**
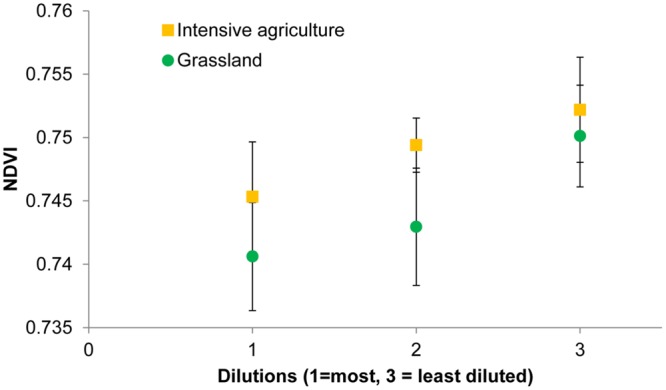
**Normalized difference vegetation index values of winter wheat plants growing in soils with different bacterial diversities as a consequence of soil dilution treatments**.

For both soils, when analyzing all 24 replicates, i.e., six blocks with bacterial species richness data and 18 blocks for which no such data are available, the mean NDVI on average declined with dilution (**Figure 4**). However, the effect of bacterial species loss via dilution is only marginally significant (*P* = 0.05) when both soils are analyzed together and not any more when the soils are analyzed separately. Four other indices changed significantly with dilution for the grassland soil (**Table 5**). Nitrogen content of the leaves was low (0.66 ± 0.014%) and not significantly affected by any of the treatments.

**Table 5:** Effect of soil dilution treatment on spectral indices for grassland and intensive monoculture soils from farm 2.

	Intensive				Grassland			
	A		B		A		B	
	*t*	*P*	*t*	*P*	*T*	*P*	*t*	*P*
NRI	-1.248	0.219	-0.508	0.614	1.293	0.204	-2.272	**0.028**
NDVI	-3.891	**<0.001**	-1.336	0.189	-1.507	0.140	2.209	0.032
RDVI	-2.266	**0.023**	-0.593	0.557	-1.965	0.057	3.776	**<0.001**
EVI	-2.902	**0.006**	0.084	0.933	-0.565	0.575	0.451	0.654
REP	-1.429	0.161	0.688	0.495	-2.395	**0.022**	3.840	**<0.001**
mREP	-1.233	0.225	-0.525	0.602	-2.402	**0.021**	3.693	**<0.001**
PRIa	-1.826	0.075	-0.181	0.857	-1.907	0.064	2.752	**0.009**
WI	-1.514	0.138	0.409	0.684	-1.832	0.075	0.172	0.865
DSWI	-2.14	0.038	0.475	0.638	-1.192	0.247	0.715	0.478
PSa	0.333	0.741	0.222	0.825	1.826	0.076	-3.159	**0.003**
ARI	0.805	0.425	1.363	0.181	2.486	**0.018**	-3.657	**<0.001**
PSRI	-0.900	0.373	-0.171	0.865	-2.151	0.038	3.690	**<0.001**

*Degrees of freedom are 40 resp 39 for intensive A resp B and 44 resp. 37 for grassland A resp B. A and B are replicate samples taken within the same field, separated by at least 30 m. Values in bold were significantly different between dilution treatments with both the linear mixed model and the non-parametric Prentice test.*

The next step in exploring the generality of the relation between bacterial species richness and spectral indices was to determine whether treatments that used inoculum from the same fields, the within-field replicates, show similar patterns of change in spectral indices with dilution. For the intensive soil the correlation is repeatable: the NDVI of intensive A showed a significant decline with dilution (**Table 5**). However, for grassland B the NDVI did not decline but tended to increase with dilution. Three indices (mREP, REP, ARI) changed significantly with dilution treatment for both grassland soils, but in opposite directions (**Table 5**). This shows that spectral reflectance can be indicative of differences in microbial communities, with the RDVI the most common responding index (**Table 5**).

## Discussion

The results were in support of the hypothesis that plants growing with microorganisms from different farms differ in spectral reflectance. Given the geographical distance between the farms different microbial communities were expected (Bates et al., 2013), with consequences for plant physiological status and thus hyperspectral reflectance. The soils from the farms may have differed also in several abiotic parameters (Hol et al., 2015a), however, these did not play a direct role here since the experiment was done with uniform sterilized soils, inoculated with field-collected microorganisms. While nutritional effects cannot be completely excluded, it seems an unlikely explanation given the fact that the differences in nutrients between farms was much smaller than the differences in nutrients between land use. Nearly all spectral indices differed between the two farms, with farm 2 presenting the qualitative higher scores. The area that is different is the red-edge and cell-structure influenced region of the spectra (Kumar et al., 2001). This region has been acknowledged as relevant to analyze plant stress, plant forage quality, nitrogen, and cell structure (Filella et al., 1995; Kumar et al., 2001; Ayala-Silva and Beyl, 2005; Ramoelo et al., 2012). The suggestion that the microorganisms from farm 2 would be qualitative better for plant growth did not translate into larger shoot biomass. Although high chlorophyll concentration may result in high radiation absorption, it does not necessarily relate to high photosynthetic rates and thus higher biomass. The discrepancy has been attributed to intraspecific differences in photosynthetic efficiency (Leopold and Kriedemann, 1975; Kumar et al., 2001). The apparent higher quality of plants growing in soil from farm 2 does match to some extent with the observation in the first phase of the experiment, where plants grew better on arable soils from farm 2 (Hol et al., 2015a).

Our second hypothesis, regarding different land use, was barely supported. Only on one of the farms difference in land use translated into different spectra indices. This is surprising, given the substantial effects that land use can have on soil biota (de Vries et al., 2013; Tsiafouli et al., 2015). The effects of land use on soil biota are heavily confounded with effects on soil abiotic conditions, a factor which was eliminated here because of the inoculation in sterilized soil approach. In general, more pathogens would be expected in soils under intensive crop rotation (Kremen and Miles, 2012), with effects on plant chemical composition, but in the present study we found no effect of land use on plant biomass, and observed no disease symptoms on the leaves. We cannot exclude non-symptomatic soil-borne diseases, only it that case we would have expected to find more differences in spectral indices. Plant-associated microorganisms could be underrepresented in this experiment following from the inoculation into sterilized soil together with the long incubation period without plants.

Finally, the last hypothesis regarding the detection of species loss via hyperspectral reflectance was partially supported. For half of the 12 soil origins a difference in one or more spectral indices was found between dilution treatments despite lack of significant differences in plant biomass. This shows that spectral reflectance is much more sensitive to microbial species loss than shoot biomass. It also shows that the loss of microorganisms does not consistently lower or improve plant chemical composition. Since dilution may result in loss of both pathogens and mutualists, depending on their relative abundance, it is not surprising that soil with different microbial communities may vary in the effect of microbial species loss on plant chemical composition. Indeed, the first phase of this experiment also showed how plant biomass could increase, decrease or not change in response to microbial species loss (Hol et al., 2015a).

For a subset of the dilution treatments a more detailed observation of the bacterial species richness could be linked with hyperspectral reflectance. Since higher NDVI values have been associated with higher photosynthesis activity and nutrient availability/presence (Filella and Penuelas, 1994; Ayala-Silva and Beyl, 2005; Mahlein et al., 2012), these results could be seen as a positive association between bacterial diversity and plant quality. We can only speculate about the mechanism behind this positive association. Microorganisms in general can help the plant with nutrient uptake, induce production of secondary metabolites, produce hormones that affect plant growth (Hol et al., 2014), all of those could change hyperspectral reflectance patterns (e.g., Kumar et al., 2001; Ayala-Silva and Beyl, 2005; Ferwerda et al., 2005; Font et al., 2005; Carvalho et al., 2013a). Soil biodiversity might benefit plants directly via changes in nutrient availability, or indirectly by offering protection against pathogens. Diverse bacterial communities are more resistant to invasions (Mallon et al., 2015) and also produce more antifungal volatiles (Hol et al., 2015b). Weidner et al. (2015) showed how diverse bacterial communities had higher enzyme activity, increased nitrogen mineralization and increased plant growth. Since we did not find any significant differences in leaf nitrogen concentrations or total shoot biomass production, a more qualitative mechanism such as changed secondary metabolites seem plausible. Most soil origins showed little effect of dilution on spectra and thus this positive relation between diversity and function is not universal. Although the effectiveness of the dilution treatments on diversity was tested by DNA sequencing for only 2 of the 12 soil origins, it is highly likely that dilution resulted in species loss, as was found in the two soil origins tested here and in several other studies (Wertz et al., 2006, 2007; Yan et al., 2015). Therefore, certainly no overall conclusions about microbial diversity in relation to plant quality (based on NDVI) can be drawn. Interestingly, the plants with the higher quality were either the non-inoculated control soils, or the high-diversity soils. This suggests a non-linear relation between diversity and function.

For application purposes it is useful to know that spectra can reflect changes in soil diversity, but there is high variation in the measurements. While the means of several spectral indices are significantly different between farms, or dilution treatments, there is too much variation in the indices for correct classification of the plants with their respective treatments. The plants’ physiology and hyperspectral reflectance are clearly sensitive to exposure to different soil microbial community compositions, yet at the same time also sensitive to several other factors. In the greenhouse this may have been a spatial effect such as light or temperature, or the different soil batches used for the blocks. These factors were not our primary focus; the crux is that the design should allow testing the main treatments of interest, as we did for microbial composition by using a highly replicated randomized block design. Our results show that hyperspectral reflectance can be very informative due to its sensitive and non-destructive nature, yet it requires a very strict experimental design with high replication and good options to filter the necessary information. This is feasible under greenhouse conditions, such as in horticulture, and in ecological experiments under controlled conditions.

Ecologists could benefit hugely from a fast, non-destructive method which provides reliable information for decisions such as continuation of an experiment, or chemical analysis of plant material. However, the current method might be too sensitive to be of value for testing microbial community composition effects outdoors. Even inoculation of winter wheat with growth-promoting bacteria did not affect spectra in the field (Adami et al., 2010), probably overshadowed by variation caused by abiotic conditions and larger organisms. It should be noted that our experiment was carried out under nutrient-poor conditions where microbial effects on plant growth might be more apparent than under high fertility conditions such as those occurring on many agricultural fields in industrialized countries. While the application of hyperspectral reflectance to detect microbial shifts might have more potential in nutrient-poor agricultural systems and in natural ecosystems, our proof of concept might give rise to further studies in order to test feasibility under nutrient-rich conditions as well. Using plants to monitor soil microbial communities could be the first step to understand, manage and protect microbial ecosystems (Bodelier, 2011). Collaboration between ecologists and remote sensing scientists improves the likelihood of a future where microbial diversity will be one of the metrics used for biodiversity monitoring from space (Skidmore et al., 2015).

## Author Contributions

WH, WHP originally formulated the idea and designed the experiments. WH performed the greenhouse experiments and did the laboratory analyses. SC performed the hyperspectral measurements. SC, WH did the statistical analyses. SC, WH wrote the manuscript. WHP provided editorial advice.

## Data Accessibility

The raw 454-pyrosequencing data have been submitted to the EMBL database (European 483 Nucleotide Archive) under accession number PRJEB5330. All plant biomass and spectra data presented here are stored in Dryad and available (Hol et al., 2016).

## Conflict of Interest Statement

The authors declare that the research was conducted in the absence of any commercial or financial relationships that could be construed as a potential conflict of interest.
