# Supplementary material for: The Potential of Hyperspectral Patterns of Winter Wheat to Detect Changes in Soil Microbial Community Composition
Source: Front Plant Sci. 2016 Jun 9;7:759. doi: 10.3389/fpls.2016.00759 (PMC4899463; doi:10.3389/fpls.2016.00759)
Supplement: Supplementary file 1 [file Image_1.PDF]

Experimental Design

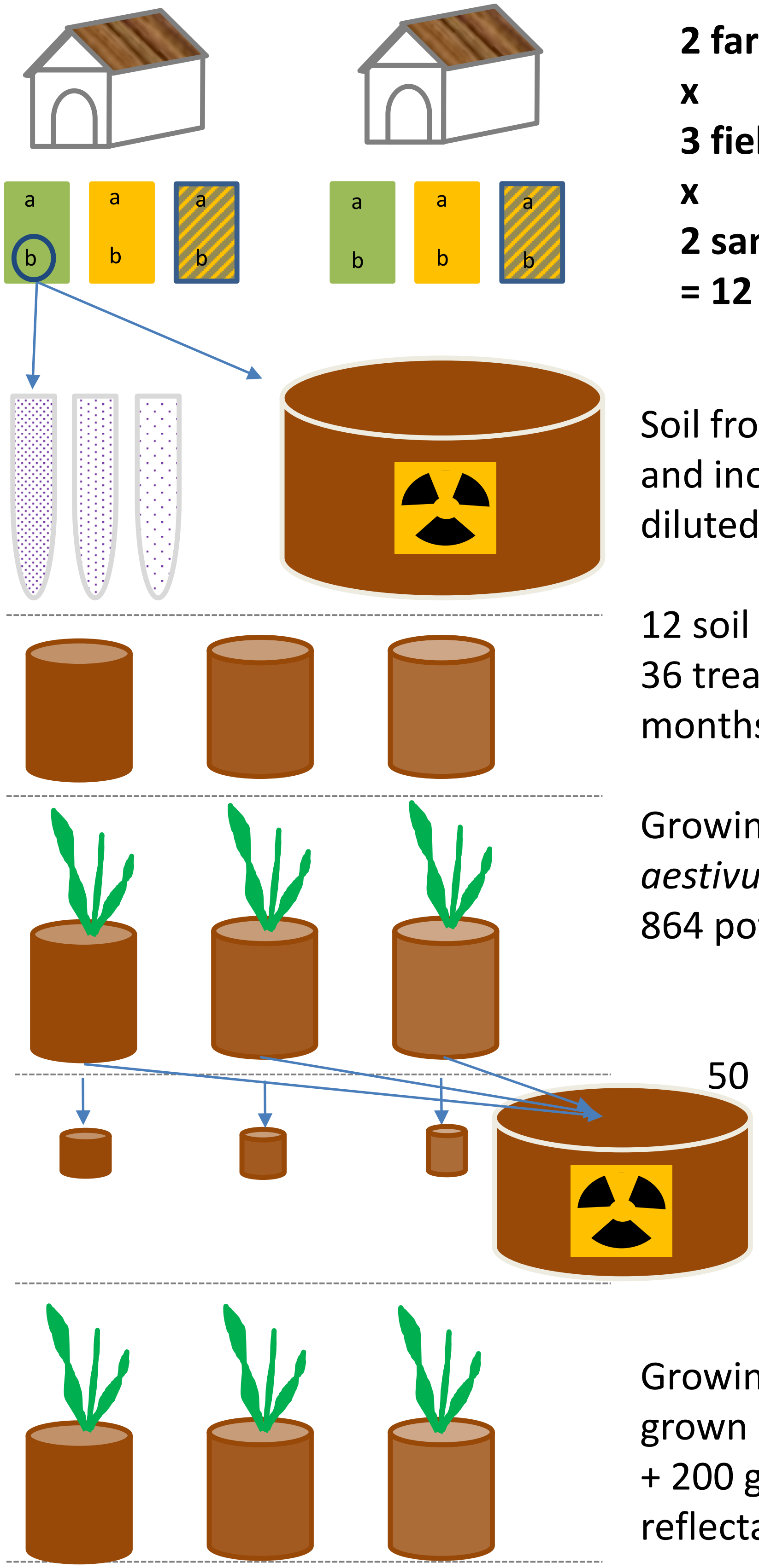

2 farms (1,2)
x
3 fields (G,I,R)
x
2 sampling locations/field (a,b)
= 12 soil origins

Soil from each origin was sterilized and inoculated with a serially diluted suspension <45µm

12 soil origins x 3 dilutions = 36 treatments were incubated for 8 months to recolonize the soil

Growing phase 1: 24 *Triticum aestivum* plants per treatment = 864 pots (Hol et al. 2015a)

50 g soil from each pot kept as inoculum. Remaining soil pooled and sterilized (25 kGray)

Growing phase 2: *T. aestivum* plants grown in a mix of 50 g soil inoculum + 200 g sterilized soil. Hyperspectral reflectance measured in week 6
